# Bilateral femoral neck fractures in an adult male following minimal trauma after a simple mechanical fall: a case report

**DOI:** 10.1186/1757-1626-2-92

**Published:** 2009-01-28

**Authors:** Asheesh Sood, Christopher Rao, Ian Holloway

**Affiliations:** 1 Northwick Park Hospital, Watford Road, Harrow, Middlesex HA1 3UJ, UK; 2 Department of Biosurgery and Surgical Technology, Imperial College London, 5 Cheerytree House, Droop Street, London W10 4EL, UK

## Abstract

**Background:**

Despite being rare there are several reports in the medical literature of bilateral femoral neck fractures in adult patients. They have been reported to have occurred following major trauma, or as a result of primary or secondary bone disease. In this case report we describe for the first time in the literature bilateral femoral neck fractures in a patient following minimal trauma after a simple mechanical fall.

**Case presentation:**

We describe the case of an 84-year-old gentleman who sustained bilateral intracapsular fractures following a simple mechanical fall. Prompt diagnosis and early surgical intervention resulted in a satisfactory outcome.

**Conclusion:**

This case highlights that in the elderly, even in the absence of primary and secondary bone disease, bilateral neck of femur fractures can occur following relatively minor trauma. Consequently, the orthopaedic surgeon, emergency physician and general practitioner should be aware of this injury, particularly when managing traumatic injuries in confused patients.

## Background

Bilateral fractures of the Neck of the Femur (NOF) have been reported to have occurred following major trauma, or as a result of primary or secondary bone disease. We describe the case of an 84-year-old gentleman who sustained bilateral intracapsular fractured NOF following a simple mechanical fall. Early diagnosis, resuscitation, surgical intervention, post-operative mobilisation and discharge; according to best practice guidelines [1]; resulted in a satisfactory outcome.

Following a review of the published literature we undertake to discuss the importance of prompt diagnosis and early surgical treatment in achieving a satisfactory outcome following this injury. Finally, we aim to discuss the implications of this case on our routine orthopaedic practice.

## Case presentation

An 84-year-old gentleman presented to the Accident and Emergency Department in the early hours of the morning after a mechanical fall down three stairs. He was an active, independent gentleman with no significant co-morbidities. He was also the full time carer for his wife who suffered from multiple sclerosis. His pre-morbid mobility was good and he did not require any walking aids.

**Figure 1:**
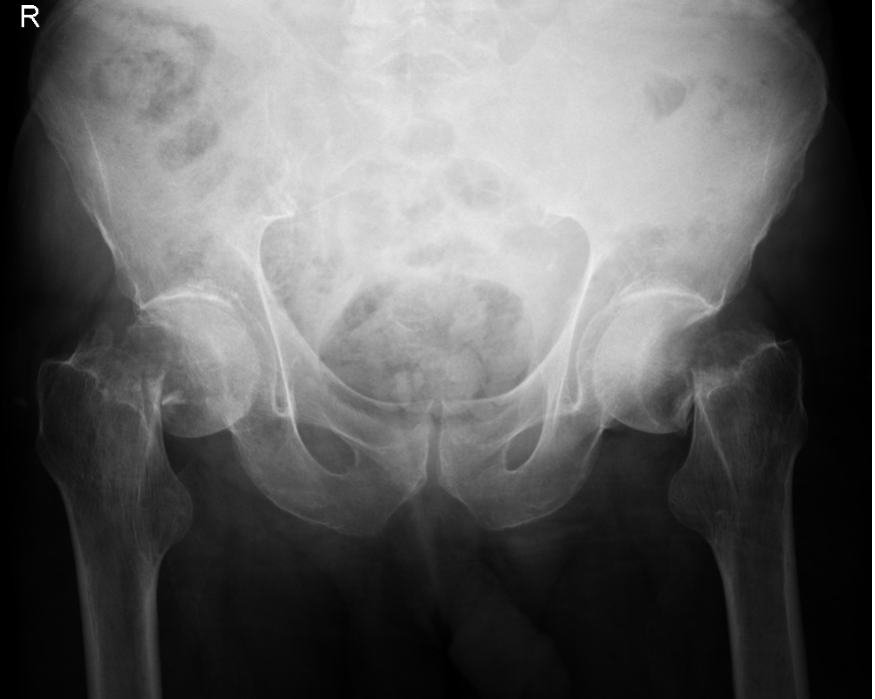
**Anterioposterior radiograph of the pelvis showing bilateral completely displaced intracapsular fractures of the neck of femur**.

He was immediately resuscitated in the Accident and Emergency Department. Clinical examination revealed external rotation of both legs and pain on passive movement of both hips. X-ray of his pelvis showed completely displaced intracapsular hip fractures on both sides [Figure 1].

**Figure 2:**
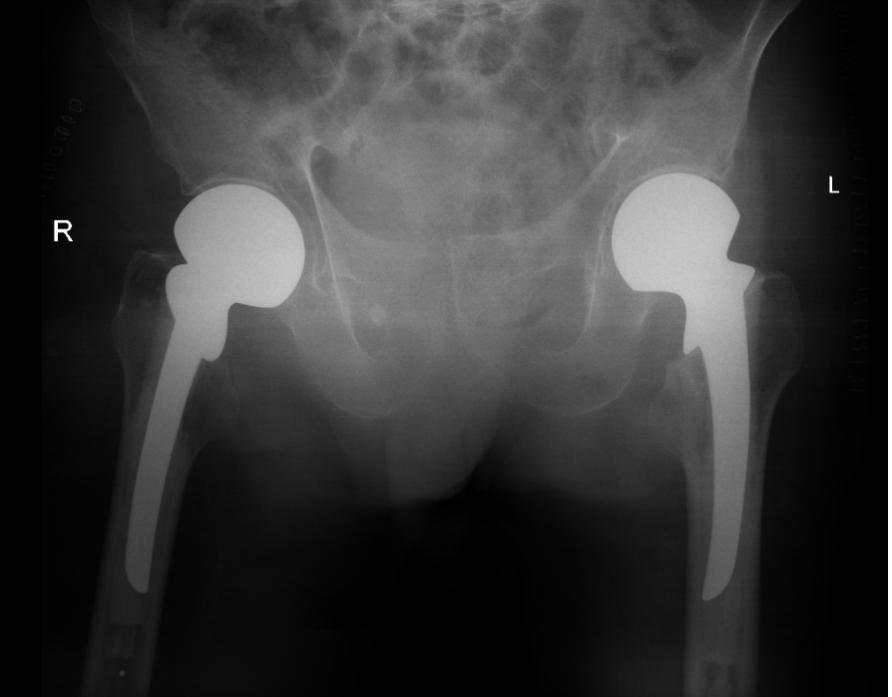
**Anterioposterior radiograph of the pelvis showing bilateral fractures of the neck of femur treated by cemented Thompson hemiarthroplasties**.

The fractures were treated by cemented hemiarthroplasty using Thompson prostheses. This was undertaken with the patient in the supine position within 24 hours of admission using an antero-lateral approach by two senior Orthopaedic surgeons [Figure 2]. He had a satisfactory post-operative recovery and was able to mobilise and fully weight-bear within 2 days of the surgery.

## Discussion

Simultaneous bilateral NOF fractures are rare. They are however, several reports in the medical literature of bilateral NOF fractures occurring as a result of primary or secondary bone disease. For example, hypocalcemia [2], osteomalacia [3], osteoporosis, renal osteodystrophy [4], radiotherapy [5] and multiple myeloma. Bilateral NOF fractures have also been reported to have occurred following persistent, sustained stress [5].

Uncomplicated trauma is a rare cause of bilateral fractured NOF [6]. There have been reports of bilateral NOF fractures after seizures secondary to epilepsy, drugs, and electrocution [6]. Intracapsular fractures of the NOF prior to the fifth decade of life usually result from severe injury [5]. Konforti et al [7] described bilateral NOF fractures in a 37-year-old gentleman crushed during a mining accident. Carrell et al [8] described the case of an 8 year old boy who sustained a bilateral neck fractures following a 25 foot fall. More recently Gunal et al [9] describe bilateral traumatic NOF fractures.

In the case we describe an otherwise fit and well gentleman who sustained bilateral NOF fractures following a low energy injury fall down three stairs. This is a common mechanism of injury which often leads to a unilateral NOF fracture. It is important to note that this patient gave a definite history of a fall which led to the injury in contrast to fatigue fractures [5] where patients describe their leg giving way followed by pain. Bilateral fractured NOF have been reported in the literature in the elderly following minimal trauma [10]. However, in this case described the patient had a background of corticosteroid induced osteoporosis, gross obesity and rheumatoid arthritis.

Guidelines for the management of hip fractures recommend that surgical intervention should be carried out within 48 hours of the fracture occurring [1]. As well as causing distress to the patient, delay in surgery is associated with increased morbidity and mortality, and a reduced chance of success and rehabilitation [11]. Surgery should be performed as soon as the medical condition of the patient allows, provided that appropriate staffing and facilities are available [11]. However, it has also been demonstrated that surgical treatment conducted as night-time emergency cases are associated with increases mortality [11].

Our management of this patient was entirely consistent with these guidelines. The fractures were diagnosed early and the patient was resuscitated appropriately. The patient was reviewed by the physicians and anaesthetists on the day of admission and optimized medically. Both hip fractures were managed by simultaneous cemented hemiarthroplasty within 24 hours of admission on a day-time trauma list. McBryde et al [12] have demonstrated the safety of simultaneous hip arthroplasties, albeit in the context of elective surgery. The operation was performed in the supine position, to avoid repositioning the patient, in order to shorten the total operating time. There is however, no evidence to support the superior efficacy of any position and we feel the surgeon should use the position with which he is most comfortable.

All hip injuries presenting to the Accident and Emergency Department in our hospital have an anterioposterior radiograph of the pelvis with both hips included as part of initial assessment. This is an important precaution as there may be an injury of the opposite hip which could easily be missed. As a result of this case, our junior surgical staff have been trained to be particularly vigilant to the possibility of bilateral NOF fractures, particularly in the very elderly, in cases where there may be primary or secondary bone disease, when the mechanism of injury is high-impact or unknown, and when patients are confused and unable to localize pain.

## Conclusion

In this case report we present a rare combination of injuries occurring simultaneously in an elderly gentleman. While a unilateral hip fracture is a very common injury managed appropriately on a regular basis by Orthopaedic surgeons, bilateral injuries of this nature presenting simultaneously can prove to be a diagnostic and therapeutic challenge. Early recognition and prompt surgical intervention can lead to good outcomes despite the severity of this injury. Bilateral anterioposterior radiographs should be taken as a matter of routine in patients presenting with suspected fractured NOF and orthopedic staff should be vigilant to the possibility of bilateral NOF fractures.

## Consent

Written informed consent was obtained from the patient for publication of this case report and accompanying images. A copy of the written consent is available for review by the Editor-in-Chief of this journal.

## Competing interests

The authors declare that they have no competing interests.

## Authors' contributions

AS and CR were responsible for drafting the case study. IH was responsible for revising it critically for important intellectual content and was the consultant ultimately responsible for managing this patient. All authors have made substantial contribution to the conception of this case report, read and approved the final version to be submitted.
